# Patterns in first and daily cigarette initiation among youth and young adults from 2002 to 2015

**DOI:** 10.1371/journal.pone.0200827

**Published:** 2018-08-10

**Authors:** Jennifer Cantrell, Morgane Bennett, Paul Mowery, Haijun Xiao, Jessica Rath, Elizabeth Hair, Donna Vallone

**Affiliations:** 1 College of Global Public Health, New York University, New York, NY, United States of America; 2 Department of Health, Behavior and Society, Johns Hopkins Bloomberg School of Public Health, Baltimore, MD, United States of America; 3 Schroeder Institute at Truth Initiative, Washington, DC, United States of America; 4 Department of Prevention and Community Health, Milken Institute School of Public Health, George Washington University, Washington, DC, United States of America; 5 Biostatistics, Inc., Atlanta, GA, United States of America; University College London, UNITED KINGDOM

## Abstract

This study’s objective was to describe long-term trends and patterns in first cigarette use (cigarette initiation) and daily cigarette use (daily initiation) among youth and young adults in the U.S. We used cross-sectional survey data from the National Survey on Drug Use and Health, 2002–2015, to estimate annual incidence of first cigarette use (N = 270,556) and first daily cigarette use (N = 373,464) for each year by age groups, race/ethnicity and gender, examining trends over time and the average annual change in initiation for each group. Several clear patterns emerged: 1) cigarette initiation and daily initiation significantly decreased over time among those aged 12–14 and 15–17 and these trends were consistent among nearly all racial/ethnic and gender subgroups; 2) among 18–21 year olds, cigarette initiation sharply increased through 2009, surpassing rates among 15–17 year olds, and sharply declined through 2015 while remaining higher than rates among the younger group, and this trend was consistent for almost all racial/ethnic subgroups; 3) daily initiation for those aged 18–21 significantly declined, and this was significant among most subgroups 4) there was no change in cigarette initiation and daily initiation for 22–25 year olds overall and most subgroups; 5) there was a significant increase in cigarette initiation for 22–25 year old Hispanics males and daily initiation for 22–25 year old males. This study provides a comprehensive look at trends in cigarette and daily initiation among U.S. youth and young adults. Despite notable declines in smoking initiation among youth and young adult populations over the last two decades, targeted prevention and policy efforts are needed for subgroups at higher risk, including young adults and Hispanic males.

## Introduction

The age of first smoking a cigarette is significantly linked to future smoking patterns and health outcomes. Early smoking initiation predicts greater likelihood of future daily [1] and heavy smoking [2], lower likelihood of quitting [3], and higher risk of lung cancer [4]. Moreover, the age of transition into daily smoking is a predictor of nicotine dependence, with those who initiate at younger ages having greater dependence than those who initiate at older ages [5]. Therefore, understanding age patterns for when people first smoke and first smoke on a daily basis can inform targeting of prevention efforts.

Trends in cigarette initiation among youth and young adults have changed over time [6]. Retrospective U.S. national data find that, among adult daily smokers, the majority of initiation occurs before age 18 [5]. However, recent longitudinal studies in the U.S. and other high-income countries have found cigarette initiation during young adulthood to be common [7–9] and increasing [10]. One national longitudinal study found that young adult initiation significantly increased from 1976 to 2005 and at a faster rate among more recent birth cohorts [10].

Changing patterns of smoking and new tobacco policy interventions, such as age 21 purchase restrictions, may differentially impact cigarette initiation risk for adolescents and young adults. While estimates of smoking prevalence are often presented for younger (12–14 years old) and older (15–17 year olds) adolescent subgroups, most studies of cigarette initiation combine these two age groups into a single “youth” subgroup. Trends among young adults are often examined with the population grouped as aged 18–25, despite significant differences between 18–21 year olds, who may be most influenced by new tobacco policies, and those aged 22–25. Studies have found dissimilar patterns of cigarette smoking uptake among those who initiate at age 19 or 20 compared with those initiating between the ages of 23 and 25 [10].

Age patterns of cigarette initiation also vary across racial and ethnic groups. Using a national sample from the Current Population Survey, Trinidad et al (2004) found that racial/ethnic minorities, specifically Hispanics, African Americans, and Asian Pacific Islanders, initiate at later ages compared with whites, with many initiating during young adulthood [11]. Studies using more recent data also support these findings [12, 13]. Despite this later age of initiation, these populations are less likely to successfully quit [14]. National data also suggest differences in cigarette initiation by gender, with females more likely to initiate smoking in late adolescence and young adulthood compared with males [15]. Evidence also suggests females are less likely to quit smoking [16] and may experience greater smoking-related health consequences [17].

Examining trends in cigarette initiation overall as well as among discrete age, racial/ethnic and gender groups is critical for evaluating how best to focus efforts to interrupt pathways to established cigarette use. This is especially important during a time of rapid technological and social change that may impact cigarette smoking patterns in unpredictable ways. New forms of targeted tobacco advertising [18–20], renewed tobacco control activity at the federal and state level [21–24], alternative tobacco products [25, 26], and broader trends in substance use [27, 28] may have significant impacts on cigarette use initiation. The objective of this study is to comprehensively describe recent national trends over time in first use of cigarettes and first daily use of cigarettes (from here referred to as cigarette initiation and daily initiation, respectively) among younger and older subgroups of adolescents and young adults and to examine these trends by race/ethnic and gender subgroups. The study is designed to identify overall and subgroup-specific metapatterns of smoking risk among young people in the United States so as to inform future tobacco surveillance and policy efforts.

## Methods

### Study sample

Study data are based on 14 waves of annual, cross-sectional survey data from the National Survey on Drug Use and Health (NSDUH) for those aged 12–25 from 2002–2015. NSDUH is a nationally representative survey that assesses drug use behaviors in the U.S. civilian, non-institutionalized population aged 12 years and older. The sample excludes members of the active-duty military and individuals in institutional group quarters (e.g., hospitals, prisons, nursing homes, treatment centers). Respondents are recruited via a multistage probability sample methodology, with 12–25 year olds and racial/ethnic minorities oversampled. Surveys are administered via in-home interviews and audio computer-assisted self-interviews (A-CASI). All data is fully anonymized.

To estimate cigarette initiation, we used 270,556 respondents aged 12–25 at risk for smoking in the year prior to their survey (i.e., excludes those who reported initiating cigarettes prior to the past year). To estimate daily initiation, we used 373,464 respondents aged 12–25 at risk for daily smoking in the year prior to their survey (i.e., excludes those who initiated daily smoking prior to the past year).

The overall weighted response rate for the 2002–2015 individual survey years ranged from 77.66% to 89.56% for respondents ages 12–17 years, and 74.45% to 83.87% for respondents ages 18–25 years [29, 30]. Due to methodological changes in the survey, comparisons for years prior to 2002 cannot be made [31]. Data were publicly available and exempt from Institutional Review Board review.

### Measures

Cigarette initiation during the previous year was measured by two items: “Have you ever smoked part or all of a cigarette?” and “Did you first smoke part or all of a cigarette in…..?” with the prior year specified. The denominator for cigarette initiation rates included never smokers and those who first smoked cigarettes during the past year. Daily initiation during the previous year was measured by, “Has there ever been a period in your life when you smoked cigarettes every day for at least 30 days?” Respondents who indicated they ever smoked every day were asked, “Did you first smoke cigarettes every day in…?” with prior year specified. The denominator for daily initiation rates included respondents who never smoked daily plus those who first smoked cigarettes daily during the past year.

We examined initiation estimates within the following groups: ages 12–14, 15–17, 18–21, and 22–25 years, race/ethnicity (whites, African Americans and Hispanics), and gender (female and male).

### Statistical analysis

SAS 9.4 was used for all analyses. We calculated annual incidence estimates for first and daily smoking by race/ethnicity, gender, and age group for each survey year. Raw point estimates and confidence intervals for all subgroups are included in Supplemental Files (see S3–S8 Figs). We examined trends over time by examining scatterplots and used weighted least squares regressions by fitting 1^st^ degree and 2^nd^ degree polynomials. A 2^nd^ degree polynomial includes terms for year and year-squared. We also calculated the average annual change in initiation per year for each subgroup by comparing the predicted initiation rate for 2002 with the predicted initiation rate for 2015. For 2^nd^ degree polynomial models the average annual change was calculated as: (2015 incidence minus 2002 incidence)/13. We also conducted sensitivity analyses for the age and racial/ethnic subgroups by fitting individual-level autoregressive models with year of interview as a categorical covariate. controlling for age. This model adjusts for serial correlation by year and has no assumptions about the shape of a fitted line. These models tested differences in the year coefficient to assess whether observed initiation rates changed significantly between 2002 and 2015.

All data were weighted [32]. The SAS survey procedures took into account NSDUH’s complex survey design. Survey weights were used to adjust for different probabilities of selection and for non-response, producing estimates representative of the U.S. population. There were no missing data on age, gender and race/ethnicity. Missing data on any smoking-related variable were 1% or less and were listwise deleted. Analyses were conducted February-March 2017.

## Results

### Cigarette initiation

**Fig 1:**
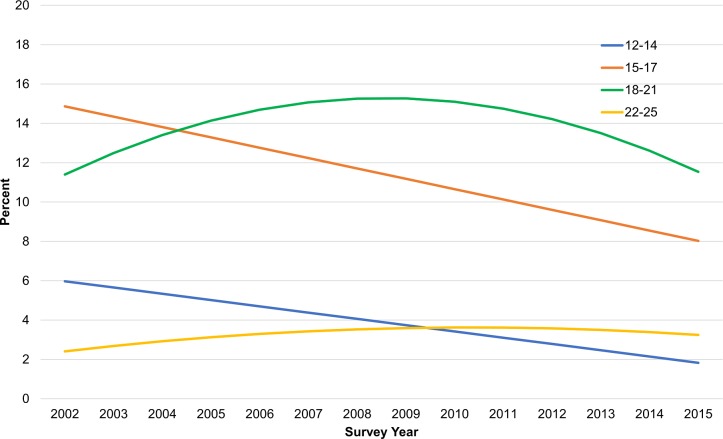
Model-based trends in the annual incidence of cigarette initiation (%), by age, males and females aged 12–25 years (source: 2002–2015 NSDUH).

**Fig 2:**
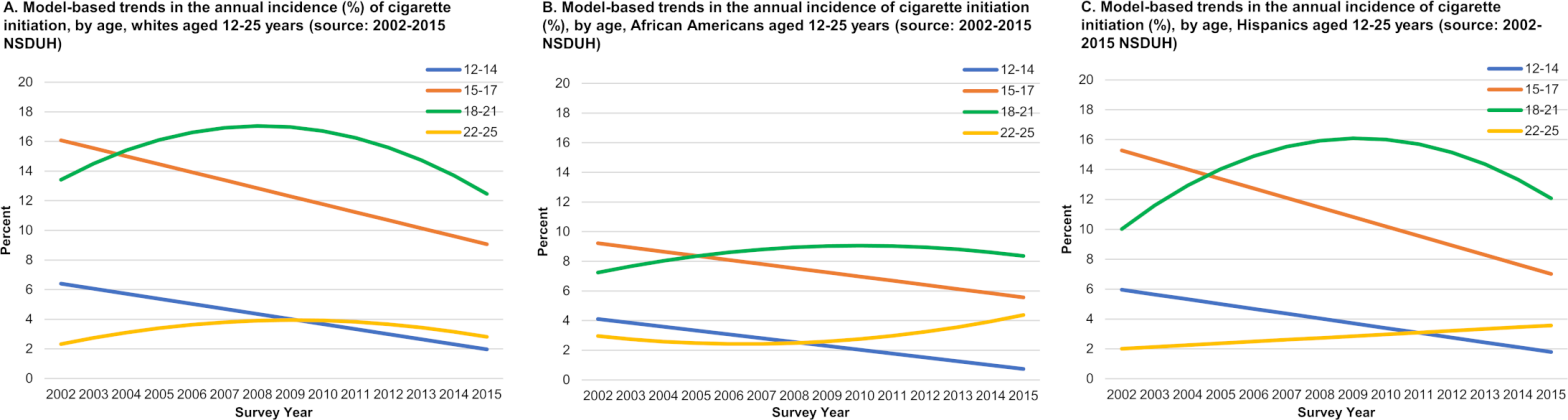
Model-based trends in the annual incidence of cigarette initiation (%), by age and race/ethnicity, males and females aged 12–25 years (source: 2002–2015 NSDUH).

Both 12–14 and 15–17 year olds experienced significant linear initiation rate declines over time, with an average annual change of -0.32 percentage points for 12–14 year-olds and -0.53 percentage points for 15–17 year-olds (Fig 1, Table 1). Relative to non-Hispanic whites, initiation rates during the time period were similar among Hispanic youth and lower among African American youth. The declines for both age groups were consistent across whites, African Americans, and Hispanics (Fig 2) and among males and females in each racial/ethnic group (see S1 Fig).

**Table 1:** Trends in the annual incidence of cigarette initiation^a^ by race/ethnicity, gender, and age (source: 2002–2015 NSDUH).

Race/ Origin	Gender	Age	Model F (p-value)^b^	R^2^	Average Annual Change (p-value)^c^
All Three Races	Overall	12–14	**183.0** **(p<0.01)**	0.94	**-0.32** **(p<0.01)**
		15–17	**109.7** **(p<0.01)**	0.90	**-0.53** **(p<0.01)**
		18–21	**22.0** **(p<0.01)**	0.80	0.01 (NS)
		22–25	2.3 (NS)	0.29	0.06 (NS)
	Male	12–14	**56.2** **(p<0.01)**	0.82	**-0.25** **(p<0.01)**
		15–17	**82.9** **(p<0.01)**	0.94	**-0.42** **(p<0.01)**
		18–21	**14.4** **(p<0.01)**	0.72	0.05 (NS)
		22–25	1.5 (NS)	0.21	0.11 (NS)
	Female	12–14	**158.8** **(p<0.01)**	0.93	**-0.38** **(p<0.01)**
		15–17	**131.9** **(p<0.01)**	0.92	**-0.58** **(p<0.01**
		18–21	**13.3** **(P<0.01)**	0.71	-0.04 (NS)
		22–25	2.9 (NS)	0.34	0.07 (NS)
Non-Hispanic White	Overall	12–14	**213.4** **(p<0.01)**	0.95	**-0.34** **(p<0.01)**
		15–17	**90.4** **(p<0.01)**	0.88	**-0.54** **(p<0.01)**
		18–21	**12.2** **(p<0.01)**	0.69	-0.07 (NS)
		22–25	2.4 (NS)	0.30	0.04 (NS)
	Male	12–14	**64.6** **(p<0.01)**	0.84	**-0.24** **(p<0.01)**
		15–17	**64.2** **(p<0.01)**	0.92	**-0.46** **(p<0.01)**
		18–21	**6.2** **(p<0.05)**	0.53	-0.07 (NS)
		22–25	1.3 (NS)	0.20	0.05 (NS)
	Female	12–14	**68.0** **(p<0.01)**	0.85	**-0.42** **(p<0.01)**
		15–17	**72.8** **(p<0.01)**	0.86	**-0.61** **(p<0.01)**
		18–21	**8.2** **(p<0.01)**	0.60	-0.10 (NS)
		22–25	2.5 (NS)	0.31	0.04 (NS)
Non-Hispanic African American	Overall	12–14	**45.4** **(p<0.01)**	0.79	**-0.26** **(p<0.01)**
		15–17	**39.2** **(p<0.01)**	0.77	**-0.28** **(p<0.01)**
		18–21	1.2 (NS)	0.18	0.09 (NS)
		22–25	1.8 (NS)	0.24	0.11 (NS)
	Male	12–14	**36.9** **(p<0.01)**	0.75	**-0.25** **(p<0.01)**
		15–17	**7.3** **(p<0.05)**	0.38	**-0.26** **(p<0.01)**
		18–21	0.36 (NS)	0.06	0.04 (NS)
		22–25	0.91 (NS)	0.14	0.15 (NS)
	Female	12–14	**13.04** **(p<0.01)**	0.70	**-0.29** **(p<0.01)**
		15–17	**14.0** **(p<0.01)**	0.54	**-0.30** **(p<0.01)**
		18–21	0.81 (NS)	0.13	0.08 (NS)
		22–25	2.98 (NS)	0.35	0.09 (NS)
Hispanic	Overall	12–14	**49.4** **(p<0.01)**	0.80	**-0.32** **(p<0.01)**
		15–17	**54.0** **(p<0.01)**	0.82	**-0.64** **(p<0.01)**
		18–21	**9.0** **(p<0.01)**	0.62	0.16 (NS)
		22–25	**18.5** **(p<0.01)**	0.61	**0.12** **(p<0.01)**
	Male	12–14	**17.54** **(p<0.01)**	0.59	**-0.29** **(p<0.01)**
		15–17	**17.86** **(p<0.01)**	0.76	**-0.37** **(p<0.01)**
		18–21	**4.96** **(p<0.05)**	0.47	0.41 (NS)
		22–25	**8.99** **(p<0.05)**	0.43	**0.25** **(p<0.05)**
	Female	12–14	**78.55** **(p<0.01)**	0.87	**-0.35** **(p<0.01)**
		15–17	**146.77** **(p<0.01)**	0.92	**-0.67** **(p<0.01)**
		18–21	**8.42** **(p<0.01)**	0.61	-0.10 (NS)
		22–25	1.36 (NS)	0.20	0.10 (NS)

^a^ Incidence rates are expressed as the percentage of never smokers who smoked 1^st^ cigarette in the year preceding survey.

^b^ The model F tests a significant trend for a 1^st^ or 2^nd^ degree polynomial for year in the model.

^c^ Average annual change is the percentage point change in initiation per year. For 1^st^ degree polynomial models (linear time trend) the average annual change is the coefficient associated with the year term. For 2^nd^ degree polynomial models the average annual change was calculated as: (2015 incidence minus 2002 incidence)/13.

Cigarette initiation rates for 18–21 year-olds overall sharply increased during the first seven years of the 2002–2015 period followed by a steep decline starting in approximately 2009 (Fig 1, Table 1). Over the full study period, the average change for 18–21 year-olds was 0.01 (p>0.05). Cigarette initiation rates for 18–21 year olds overall surpassed rates among 15–17 year olds in 2004; across subgroups, 18–21 year old rates surpassed 15–17 year olds in the years ranging from 2004 to 2006. Trends varied by race/ethnicity and gender for this age group (Fig 1). Among non-Hispanic whites, rates increased and then decreased, ending at about the same initiation rate in 2015 that was observed in 2002 (p>0.05). The same trend was observed for 18–21 year-old African Americans, although the sharp increase followed by a decrease was not as great and rates overall were lower. Among Hispanics, cigarette initiation rates increased during 2002–2009 and then decreased, resulting in no significant change over the entire time period (p>0.05) The increase during 2002–2009 was more dramatic among Hispanic males. For this group, initiation rates reached a high of 18.4% in 2010 before beginning to decline through 2015. Initiation rates among Hispanic females aged 18–21 years followed a similar pattern as observed for Hispanic males, although the increase and decrease in rates was not as great (see S1 Fig).

Cigarette initiation rates increased slightly through 2010 and then declined for 22–25 year olds, resulting in no significant change overall during this time period (Fig 1, Table 1). For this age group, time trends were similar for whites and African Americans overall (Fig 2) and for males and females within each racial/ethnic group (see S1 Fig). Hispanics aged 22–25 experienced a significant linear increase of 0.12 percentage points (p<0.01), resulting in an increase in initiation of about 1.6 percentage points over the study period. The increase among 22–25 year old Hispanics was driven by Hispanic males who experienced a linear and significant annual increase of 0.25 percentage points (p<0.01). Hispanic females aged 22–25 years experienced no change over time (see S1 Fig).

Trends for cigarette initiation among the age groups overall and by race/ethnicity in the sensitivity analyses (see S1 Table) were similar to those above, although there were some minor differences in p-values for some subgroups.

### Daily initiation

**Fig 3:**
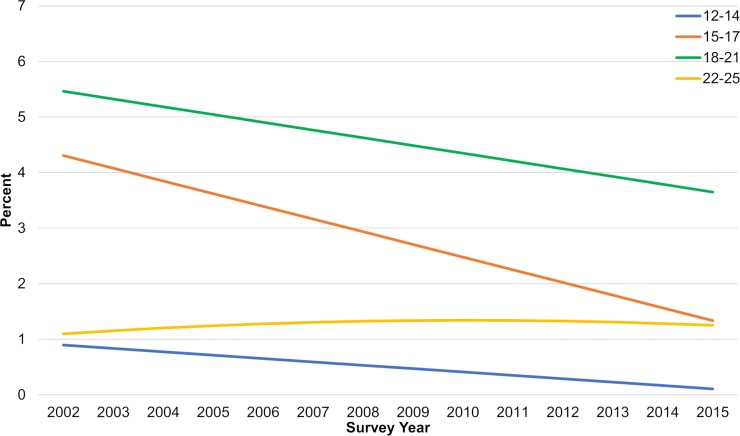
Model-based trends in the annual incidence of daily cigarette initiation (%), by age, males and females aged 12–25 years (source: 2002–2015 NSDUH).

**Fig 4:**
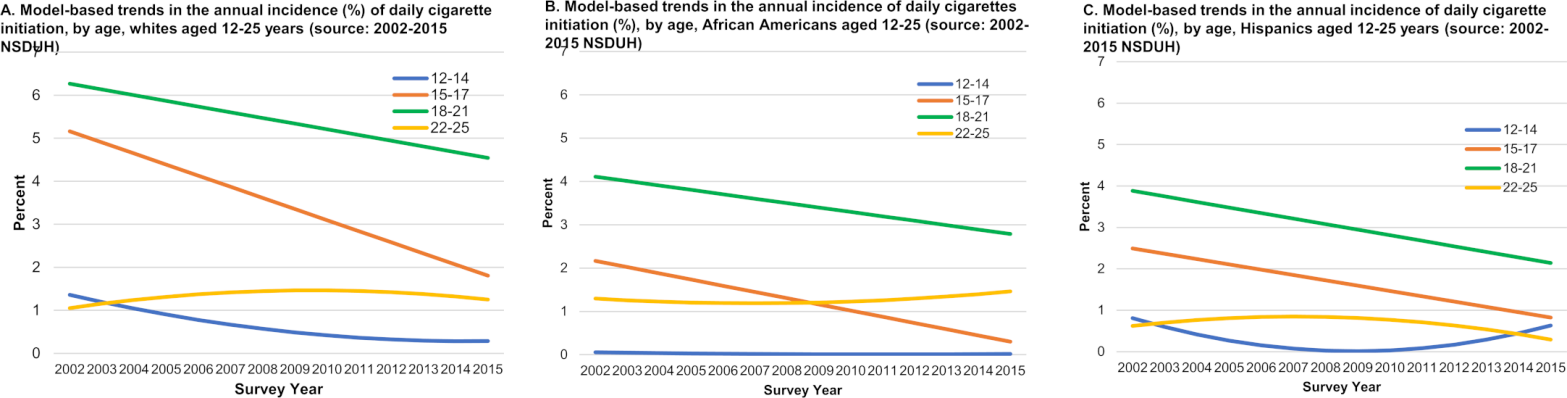
Model-based trends in the annual incidence of daily cigarettes initiation (%), by age and race/ethnicity, males and females aged 12–25 years (source: 2002–2015 NSDUH).

Daily initiation rates declined significantly over time for 12–14, 15–17 and 18–21 year-old age groups (Fig 3, Table 2) and the declines were consistent for both males and females. Subgroup sizes by race/ethnicity were too small to examine trends among 12–14 year olds. Trends among 15–17 and 18–21 year-olds for each racial/ethnic group overall (Fig 4) and by gender (see S2 Fig) were generally consistent with the overall trends with some exceptions: 15–17 year-old African American females, 18–21 year old African American and non-Hispanic white males, and 18–21 year old Hispanic females experienced no significant change. Almost all the subgroups that experienced no change had much lower initiation rates during the study period compared with daily rates overall.

**Table 2:** Time trends for incidence of daily cigarette initiation^a^ by race/ethnicity, gender, and age (source: 2002–2015 NSDUH).

Race/ Origin	Gender	Age	Model F (p-value)^b^^/^	R^2^	Average Annual) Change (p-value)^c^
All Three Races	Both	12–14	**60.7** **(p<0.01)**	0.84	**-0.06** **(p<0.01)**
		15–17	**71.4** **(p<0.01)**	0.86	**-0.23** **(p<0.01)**
		18–21	**16.3** **(p<0.01)**	0.58	**-0.14** **(p<0.01)**
		22–25	0.9 (NS)	0.14	0.01 (NS)
	Male	12–14	**48.1** **(p<0.01)**	0.80	**-0.05** **(p<0.01)**
		15–17	**40.3** **(p<0.01)**	0.77	**-0.21** **(p<0.01)**
		18–21	**5.6** **(p<0.05)**	0.32	**-0.12** **(p<0.01)**
		22–25	2.6 (NS)	0.32	**0.04** **(p<0.05)**
	Female	12–14	**53.5** **(p<0.01)**	0.82	**-0.08** **(p<0.01)**
		15–17	**66.2** **(p<0.01)**	0.85	**-0.24** **(p<0.01)**
		18–21	**21.5** **(p<0.01)**	0.64	**-0.15** **(p<0.01)**
		22–25	0.7 (NS)	0.12	-0.02 (NS)
Non-Hispanic White	Both	12–14	¶	¶	¶
		15–17	**59.4** **(p<0.01)**	0.83	**-0.26** **(p<0.01)**
		18–21	**7.2** **(p<0.05)**	0.38	**-0.13** **(p<0.05)**
		22–25	1.7 (NS)	0.23	0.02 (NS)
	Male	12–14	¶	¶	¶
		15–17	**40.5** **(p<0.01)**	0.77	**-0.21** **(p<0.01)**
		18–21	5.2 (NS)	0.49	-0.12 (NS)
		22–25	3.2 (NS)	0.37	0.05 (NS)
	Female	12–14	¶	¶	¶
		15–17	**55.7** **(p<0.01)**	0.82	**-0.30** **(p<0.01)**
		18–21	**7.3** **(p<0.05)**	0.38	**-0.13** **(p<0.05)**
		22–25	0.5 (NS)	0.09	-0.02 (NS)
Non-Hispanic African American	Both	12–14	¶	¶	¶
		15–17	**49.1** **(p<0.01)**	0.80	**-0.14** **(p<0.01)**
		18–21	**6.8** **(p<0.05)**	0.36	**-0.10** **(p<0.05)**
		22–25	0.21 (NS)	0.04	0.01 (NS)
	Male	12–14	¶	¶	¶
		15–17	3.2 (NS)	0.37	**-0.15** **(p<0.01)**
		18–21	0.62 (NS)	0.10	-0.002 (NS)
		22–25	0.87 (NS)	0.14	-0.02 (NS)
	Female	12–14	¶	¶	¶
		15–17	2.8 (NS)	0.33	-0.06 (NS)
		18–21	**42.0** **(p<0.01)**	0.28	**-0.22** **(p<0.01)**
		22–25	0.1 (NS)	0.02	0.01 (NS)
Hispanic	Both	12–14	¶	¶	¶
		15–17	**10.8** **(p<0.01)**	0.47	**-0.13** **(p<0.01)**
		18–21	**12.7** **(p<0.01)**	0.51	**-0.13** **(p<0.01)**
		22–25	2.1 (NS)	0.27	-0.03 (NS)
	Male	12–14	¶	¶	¶
		15–17	**8.9** **(p<0.05)**	0.43	**-0.15** **(p<0.05)**
		18–21	**8.7** **(p<0.05)**	0.42	**-0.21** **(p<0.05)**
		22–25	3.8 (NS)	0.43	-0.03 (NS)
	Female	12–14	¶	¶	¶
		15–17	3.2 (NS)	0.36	**-0.11** **(p<0.05)**
		18–21	2.0 (NS)	0.27	-0.07 (NS)
		22–25	1.3 (NS)	0.19	-0.04 (NS)

^a^Incidence rates are expressed as the percentage of persons who first smoked cigarettes daily in year preceding the NSDUH survey.

^b^ The model F tests a significant trend for a 1^st^ or 2^nd^ degree polynomial for year in the model.

^c^ Average annual change is the percentage point change in initiation per year. For 1^st^ degree polynomial models (linear time trend) the average annual change is the coefficient associated with the year term. For 2^nd^ degree polynomial models the average annual change was calculated as: (2015 incidence minus 2002 incidence)/13.

¶ Not reported due to large relative standard error.

Rates of daily initiation for 22–25 year olds over time (Fig 3, Table 2) were flat overall, among females, and for each racial/ethnic and gender subgroup (Fig 4 and S2 Fig). However, rates were generally very low and less than 3% each year for each group. There was a small but statistically significant increase in daily initiation among males overall (annual rate increase = 0.04 percentage points, p<0.05).

Trends for daily initiation among the age groups overall and by race/ethnicity in the sensitivity analyses (see S1 Table) were similar to those above, although there were some minor differences in p-values for some subgroups.

## Discussion

Findings from this study document trends in cigarette initiation and daily initiation among youth and young adults overall, by age, gender, and race/ethnicity. Among youth aged 12–17, most initiation occurred during the ages of 15–17. However, we found significant declines in cigarette initiation from 2002–2015 for both 12–14 and 15–17 year old adolescents. In contrast, cigarette initiation rates among 18–21 year-olds surpassed those of 15–17 year olds during the study period and have remained higher, even with declines seen among both groups since approximately 2009. Cigarette initiation rates remained unchanged among 22–25 year-olds, although rates among Hispanic males of this age group increased significantly.

For daily initiation, rates for 18–21 year-olds were higher than 15–17 year-olds over the study period. However, declines in rates were found among 12–14, 15–17, and 18–21 year-olds. Daily initiation rates among 22–25 year-olds were flat, with the exception of an increase among males overall. There were no other significant rate increases among 22–25 year-olds.

These trends should be considered in the context of cigarette smoking prevalence rates over time. National data from NSDUH indicate that both adolescents and young adults have experienced significant declines in past 30-day (current) and daily smoking prevalence from 2002 to 2015 [33]. However, the decline in both current and daily cigarette smoking was slower for 18–25 year olds and approximately half that of the 12–17 year old decline [33]. Further, since approximately 2012, the rate of decline for current and daily smoking has increased substantially for the younger group but has remained relatively flat among young adults, particularly for daily smoking [33]. Such trends reflect the consistent declines in youth initiation that we see among the 12–14 and 15–17 year olds in this study [33]. The more variable rates of first initiation among 18–21 year olds and the flat rates in cigarette initiation and daily initiation among the 22–25 year olds may be key contributing factors to the slower declines in prevalence in young adults relative to youth.

Declines in smoking among youth and more variable rates among young adults may also be a result of broader trends in alternative tobacco product use among these populations. National data from 2002 to 2011 demonstrate that cigar initiation and past 30-day cigar prevalence significantly declined among 12–17 year olds but both initiation and prevalence remained flat among 18–25 year olds.[5, 34]. Researchers suggest that trends in cigar use among young adults is consistent with a pattern of using inexpensive cigarillos and little cigars to initiate and, eventually, maintain cigarette use [34]. If cigar use among young people leads to cigarette initiation, we might expect cigarette initiation patterns similar to those we see in this study, such that cigarette initiation would decline over time among the 12–17 year olds and remain relatively flat among 18–25 year olds.

Other trends in alternative tobacco product use may also be contributing to rates of cigarette initiation. However, this is difficult to assess over the 2002–2015 time period as national surveys did not ask about products such as hookah and e-cigarettes until approximately 2010 and 2011 and many national surveys still do not include appropriate questions. While data are still limited, research suggests that age of initiation for these products is distinctly different from that of cigarettes. Studies suggest hookah initiation may begin at approximately age 19 and e-cigarettes age 17.5, compared with age 14 or 15 for cigarettes [35–39]. Given the later age initiation profiles of these products, their impact would likely be seen most strongly on young adult tobacco use patterns. If initiation and use of these products during late adolescence and early young adulthood leads to cigarette initiation, such patterns may contribute to flat or rising rates of cigarette initiation among young adults. However, to the extent that young adults who would have smoked cigarettes anyway may use alternative tobacco products instead, use of these products may reduce or replace cigarette initiation. Given limited data, however, determining the impact of these products on cigarette initiation over the time period of this study is not possible here.

Below we further discuss the main trends identified among key subgroups.

### Declines in cigarette initiation and daily initiation among youth aged 12–17 years

Declines in cigarette and daily initiation among the younger age groups are consistent with declines in smoking documented in existing literature and described above [33, 40, 41], as well as prior research on initiation. In an examination of daily initiation between 2006 and 2013, Thompson et. al. (2017) found significant declines among those aged 12–17 years, although declines among 12–17 year old African American males and females and among Hispanic females were not as great as declines among their white counterparts [42]. Our findings regarding daily initiation are consistent with Thompson. In our study, while rates of decline for these minority groups were smaller than for non-Hispanic whites, rates overall were approximately half that of white males and females of the same age and remained very low (under 1–2%) over the study period.

In addition to changing patterns of alternative tobacco product use discussed earlier, the declines in cigarette initiation and daily initiation among youth may be the result of multicomponent tobacco prevention initiatives over the past decade. Research has demonstrated that increased state tobacco control expenditures from 2002 to 2008 contributed to a significant decline in youth smoking susceptibility, first initiation, and current and established smoking [43] while new smoke-free air laws contributed to declines in smoking susceptibility, current and established smoking among youth. In addition, higher cigarette taxes, increasingly implemented during the past decade, were also associated with declines in current smoking among youth [43]. Further, the 1990’s and 2000’s saw policies that limited youth access proliferating rapidly across states, with studies showing effectiveness in reducing youth smoking prevalence [44]. Industry marketing to young people under the age of 18, a well-established cause of youth smoking [5, 45], was also significantly curtailed after implementation of the 1998 Master Settlement Agreement. Finally, national countertobacco mass media campaigns, such as the original truth® campaign in the early- and mid-2000s targeted 12–17 year olds and was found to have prevented 450,000 youth from initiating smoking during that time [46], while the more recent FDA-sponsored Real Cost campaign was found to have prevented nearly 350,000 youth aged 11–18 from smoking initiation from 2014 to 2016 [47].

### Cigarette initiation delayed while daily initiation declined among 18–21 year olds

We found that cigarette initiation was higher among 18–21 year olds than among youth for the period from approximately 2004 to 2015. This suggests that cigarette initiation is being delayed until young adulthood, with the highest risk of initiation occurring among young adults aged 18–21. Our findings suggest a continuation of trends identified in other studies, which have demonstrated delayed initiation and an increase in cigarette uptake among young adults [10, 48]. In a national longitudinal analysis in the U.S. among youth who had not initiated by senior year of high school, smoking uptake was most likely to occur at ages 19–20 [10]. Findings from this and other studies may be part of a broader trend of declining initiation of risky health-related behavior during adolescence [27, 49–51], resulting in a later age of initiation for multiple forms of substance use [52]. Given that cessation rates are higher and tobacco-related disease lower for later initiators [12, 13, 53], delays in adolescent uptake can reduce the harms of smoking. However, these trends point to the need to improve and enforce policies to reduce first initiation among the young adult population. Raising the minimum age of purchase to age 21 could be particularly effective. Simulation models based on national data find that such policies could reduce initiation for this age group and result in 4.2 million fewer years of life lost for those born between 2000 and 2019 [54].

While cigarette initiation rates were rising and then falling for 18–21 year-olds during the study period, daily initiation was decreasing among this age group—a finding that emphasizes the need to look at both forms of initiation. The decline in daily initiation is likely the result of the significant and consistent decline in smoking prevalence [40] and cigarette initiation among the younger 12–17 year old age groups from the 1990s to the present. With a declining population initiating at ages 12–17, daily smoking initiation at age 18–21 would be expected to decline during the study period given fewer youth who have ever smoked. Reductions in heavy daily smoking in the population overall [41, 51] and the associated changes in social norms around heavy use may also contribute to declining daily initiation rates.

In addition to possible influences by alternative tobacco product use patterns described earlier, the trends identified in this study may also be influenced by industry marketing that fosters experimental or first smoking uptake among young adults through discounts and promotions of premium brands. Young adult experimental and occasional smokers are more likely than regular smokers to use premium brands [55]. From 2002 to 2011, the market share of premium brands in the U.S. increased during a time in which the industry was increasingly using price reductions to keep premium brands competitive with discount brands [55–59]. These price reduction strategies may be particularly effective at encouraging young people to smoke as youth are typically more price sensitive [5]. Such trends could result in increased cigarette initiation among young adults. Without increased targeting of price promotions aimed at potential daily smokers–a group more likely to use discount brands [55]–one would not expect to see a similar increase in daily initiation among this age group.

### Flat cigarette initiation and daily initiation rates for 22–25 year olds

As may be the case with 18–21 year old first smokers, the lack of a decline in cigarette initiation rates among 22–25 year-olds may indicate increased targeting of young adult non-smokers with price promotions. Similarly, flat daily initiation rates, while low, likely reflects a portion of the 18–21 year-old cigarette initiators transitioning to daily initiation and greater dependence as they age. Recent longitudinal research examining progression to patterns of established smoking among young adults finds that those who escalate rapidly to daily use stabilize by age 21, while a proportion of occasional users stabilize at daily use by age 23 [60]. Given that an increased cohort of 18–21 year-olds initiated first smoking from 2002 to 2009, a portion of that cohort would have transitioned to daily initiation as they aged—thus potentially contributing to flat daily initiation rates among the 22–25 year old group.

### Rapid reversal or faster declines for most groups after 2009

Rates of cigarette initiation and daily initiation among all age groups may have been higher by 2015 if not for the increased tobacco control activity that occurred starting in 2009. In post-hoc analyses, we found that all age groups experienced either a post-2009 reversal in the direction of the trend toward a decline or a faster rate of decline. These trends were largely consistent across racial/ethnic and gender subgroups and occurred rapidly. Such a consistent trend across all groups is very likely to have been influenced by events at the national level, which began in 2009 and were among the most significant anti-tobacco control initiatives since the early part of the decade [61]. The Family Smoking Prevention and Tobacco Control Act was signed into law in June of 2009, which resulted in restrictions on cigarette marketing by prohibiting the use of certain types of industry promotions and retail marketing methods [24]. Additionally, the largest federal cigarette excise tax increase in history also occurred in 2009, leading to a 158% increase in federal taxes from 0.39 cents to $1.01 for a pack of cigarettes [62]. Van Hasselt et. al. (2015) examined smoking behavior among youth and young adults before and after the tax increase in April of 2009 and found that the tax increase significantly reduced youth smoking initiation, as well as current smoking and frequency of smoking among youth and young adults [63]. The tax was associated with a marginally significant negative trend in initiation for young adults (p = .0532) [63]. Further, the truth® FinishIt campaign starting in 2014 was the first national prevention campaign targeting young adults and was found to have prevented over 300,000 15–21 year olds from smoking over the course of a year [64].

### Some race/ethnic subgroups at increased risk for first and/or daily smoking initiation

#### Hispanics

Among all three racial/ethnic subgroups, only Hispanic males aged 22–25 years experienced an increase in initiation rates, and this occurred for cigarette initiation. Cigarette initiation rates increased and then declined among 18–21 year old Hispanic males and females during the study period, while daily initiation rates were flat among 22–25 year old Hispanic males and females. Given declining initiation among 12–17 year-old Hispanics, increases in cigarette initiation among adult males may be partially due to late onset initiation.

Changing immigration and demographic patterns in the U.S. may also play a role in shaping cigarette smoking trends among Hispanics. Prior research suggests U.S.-born Hispanic young adults have higher smoking prevalence than foreign-born Hispanic young adults, even after adjusting for socioeconomic status, and the former group also demonstrates predominant patterns of light and occasional smoking [65]. The proportion of Hispanics who are U.S.-born has increased since 2000, while the proportion of foreign-born Hispanics has declined [66], thus potentially contributing to increases in first smoking initiation among this U.S.-born population. Additionally, the proportion of Hispanics living in the Southern U.S. has been increasing over time, possibly exposing this group to social norms more favorable to tobacco use and less strict tobacco control policies present in this region of the country [66]. While likely playing a lesser role, the increase in Puerto Ricans moving to the U.S. mainland [67] may have contributed to these trends. Puerto Ricans are the second largest group of U.S. Hispanics and have high rates of smoking [68, 69]. A combination of these demographic changes may all contribute to increasing first smoking initiation among 22–25 year old Hispanic males and flat rates among the other young adult Hispanic subgroups.

Rising and stalled initiation rates are a concern. While later initiation may be health protective for Hispanics as it is with whites, there is limited research examining how early- versus late-smoking onset may influence longer-term cessation and health-related outcomes for varying groups of Hispanics. Increased cigarette initiation among young adult groups may lead to long-term patterns of non-daily smoking, which does not translate to higher rates of successful quitting for Hispanics as expected compared with non-Hispanic whites [14, 70].

#### African Americans

Overall, cigarette initiation and daily initiation rates for African American young adults were mostly lower than among whites from 2002–2015, a finding that supports other cross-sectional and longitudinal studies [10, 13, 71]. The results of the current study also support prior research finding African Americans have a later age of smoking onset compared with whites [13, 42]. However, lower cigarette initiation rates relative to whites do not take into account use of other combustible products. While initiation rates overall may be lower among African Americans and young adult onset more common compared with whites, a greater proportion of African Americans also initiate with marijuana before tobacco, and this proportion has been increasing over time [72]. Thus, African American young adults who are initiating cigarettes in young adulthood may already be marijuana users and thus initiating into dual use of marijuana and cigarettes. Further, the gap between African American and white young adult smoking prevalence is less pronounced when considering use of cigars, marijuana, and cigarettes versus just cigarettes alone [72].

Our findings for African American trends over time differ somewhat from Thompson’s, which indicated an increase in daily initiation among successive annual cohorts of black males age 18–25 [42] while we found no change for 18–21 year old and 22–25 year old males. Differences are likely due to the more recent study period used in the current study and the continued effects of the post-2009 decrease in rates. The lack of a decline in initiation among young adult African American smokers in the current study is a concern given that late-onset African American smokers have the lowest cessation rates compared with their early-onset counterparts and with early- and late-onset white smokers [13]. Evidence suggests late onset smoking among African Americans is also not protective in terms of mortality as it is for white smokers [13].

#### Whites

Trends among white young adults differed compared with African Americans and Hispanics. Cigarette initiation rates among 18–21 year olds were much higher among whites overall compared with African Americans, and slightly higher compared with Hispanics. Both white and Hispanic males and females aged 18–21 years experienced increasing followed by decreasing rates during the study period compared with relatively flat rates for African American males and females. This tracks earlier trends in smoking prevalence, which showed somewhat higher current smoker prevalence overall among white young adults compared with African American young adults, but greater declines for whites from the late 1990s through 2005 [73]. The trends observed in the current study reflect similar trends for health risk behaviors across racial/ethnic groups. Data over the last decade find improved trends over time in marijuana and alcohol use among white 12^th^ graders compared with trends among their African American and Hispanic counterparts [51]. Whites aged 18–21 also experienced declines in daily initiation that were greater in magnitude than African Americans, which is consistent with Thompson [42].

### Limitations

Limitations of this study include the use of self-reported data for cigarette use and possible differences in reporting by age, given youth are often interviewed in their parents’ home. However, there is little evidence of underreporting of smoking among young people [74] and level of privacy in NSDUH interviews is high. State tobacco control policy variables, region, country of origin, and nativity variables could not be used to examine trends identified in this study as the NSDUH restricted data files needed to conduct these analyses have not been made available. Due to smaller sample sizes, we could not model trends over time for daily initiation among 12–14 year olds as estimates were unstable. Further,we acknowledge debates regarding measures of any first cigarette use and the extent to which these measures predict subsequent regular smoking later in life [75–77]; however, the measure used in this study has been found to predict regular smoking [78]. We also did not evaluate how changes in alternative tobacco products, such as e-cigarettes, hookah, and cigars may have influenced cigarette trends as NSDUH does not collect data on several of these products and these additional analyses were outside the scope of this paper.

Strengths of this study include the large nationally representative sample and the construction of rates using data obtained on initiation during the prior year based on data from the subsequent year. This approach minimizes recall bias and provides timely surveillance on the incidence of cigarette smoking patterns among current youth and young adult populations. Additionally, NSDUH sampling covers the entire youth population in the U.S., including both those enrolled and those not enrolled in school. This is of particular importance when assessing substance use as prevalence of use differs between those enrolled in school and those who have dropped out [79]. Finally, by examining initiation rates among discrete age groups of older and younger youth (i.e., 12–14 and 15–17) and older and younger young adults (i.e., 18–21 and 22–25), we are better able to identify specific patterns in groups of young people who differ with regard to lifestyle, levels of independence, identify formation, and legal access to tobacco and alcohol.

### Conclusions

Detailed understanding of trends in cigarette initiation and daily initiation is critical for effectively targeting prevention efforts to those most at risk. Despite notable success in declining smoking initiation rates across youth and young adult populations, targeted prevention and policy efforts are needed for specific age and racial/ethnic subgroups at higher risk. Results from this study are consistent with other research demonstrating that smoking onset is becoming more concentrated during young adulthood. Prevention and cessation efforts are needed to address rising rates of daily initiation among 22–25 year old males. Further, young adult Hispanic males aged 22–25 were found to be at increased risk for first cigarette use, and African American young adults have experienced low but stalled rates of cigarette initiation and daily initiation for over a decade. Further research is needed to illuminate how population-level changes in demographic trends and non-cigarette substance use behaviors may influence cigarette initiation among these groups. Future longitudinal studies and improved surveillance are critical for understanding how shifts in the tobacco product marketplace, tobacco policies, and tobacco marketing environment continue to shape trends in cigarette initiation among young people. Given declining smoking prevalence among adults, appropriately targeted policies and interventions to reduce initiation among youth and young adults may offer the best prospects for a tobacco endgame.

## Supporting information

S1 Fig Model-based trends in the annual incidence of cigarette initiation (%), by age, gender, and race/ethnicity (source: 2002–2015 NSDUH). (PDF)

S2 Fig Model-based trends in the annual incidence of daily cigarette initiation (%), by age, gender, and race/ethnicity (source: 2002–2015 NSDUH). (PDF)

S3 Fig Raw annual cigarette initiation rates (%) and confidence intervals, by age, males and females aged 12–25 years (source: 2002–2015 NSDUH). (PDF)

S4 Fig Raw annual cigarette initiation rates (%) and confidence intervals, by age and race/ethnicity, males and females aged 12–25 years (source: 2002–2015 NSDUH). (PDF)

S5 Fig Raw annual cigarette initiation rates (%) and confidence intervals, by age, gender, and race/ethnicity (source: 2002–2015 NSDUH). (PDF)

S6 Fig Raw annual daily cigarette initiation rates (%) and confidence intervals, by age, males and females aged 12–25 years (source: 2002–2015 NSDUH). (PDF)

S7 Fig Raw annual daily cigarette initiation rates (%) and confidence intervals, by age and race/ethnicity, males and females aged 12–25 years (source: 2002–2015 NSDUH). (PDF)

S8 Fig Raw annual daily cigarette initiation rates (%) and confidence intervals, by age, gender, and race/ethnicity (source: 2002–2015 NSDUH). (PDF)

S1 Table Autoregressive models of cigarette initiation and daily initiation among age, gender and racial/ethnic groups (source: 2002–2015 NSDUH). (PDF)
