# Supplementary material for: Patterns in first and daily cigarette initiation among youth and young adults from 2002 to 2015
Source: PLoS One. 2018 Aug 10;13(8):e0200827. doi: 10.1371/journal.pone.0200827 (PMC6086419; doi:10.1371/journal.pone.0200827)

S1 Fig. Model-based trends in the annual incidence of cigarette initiation (%), by age, gender, and race/ethnicity (source: 2002-2015 NSDUH)

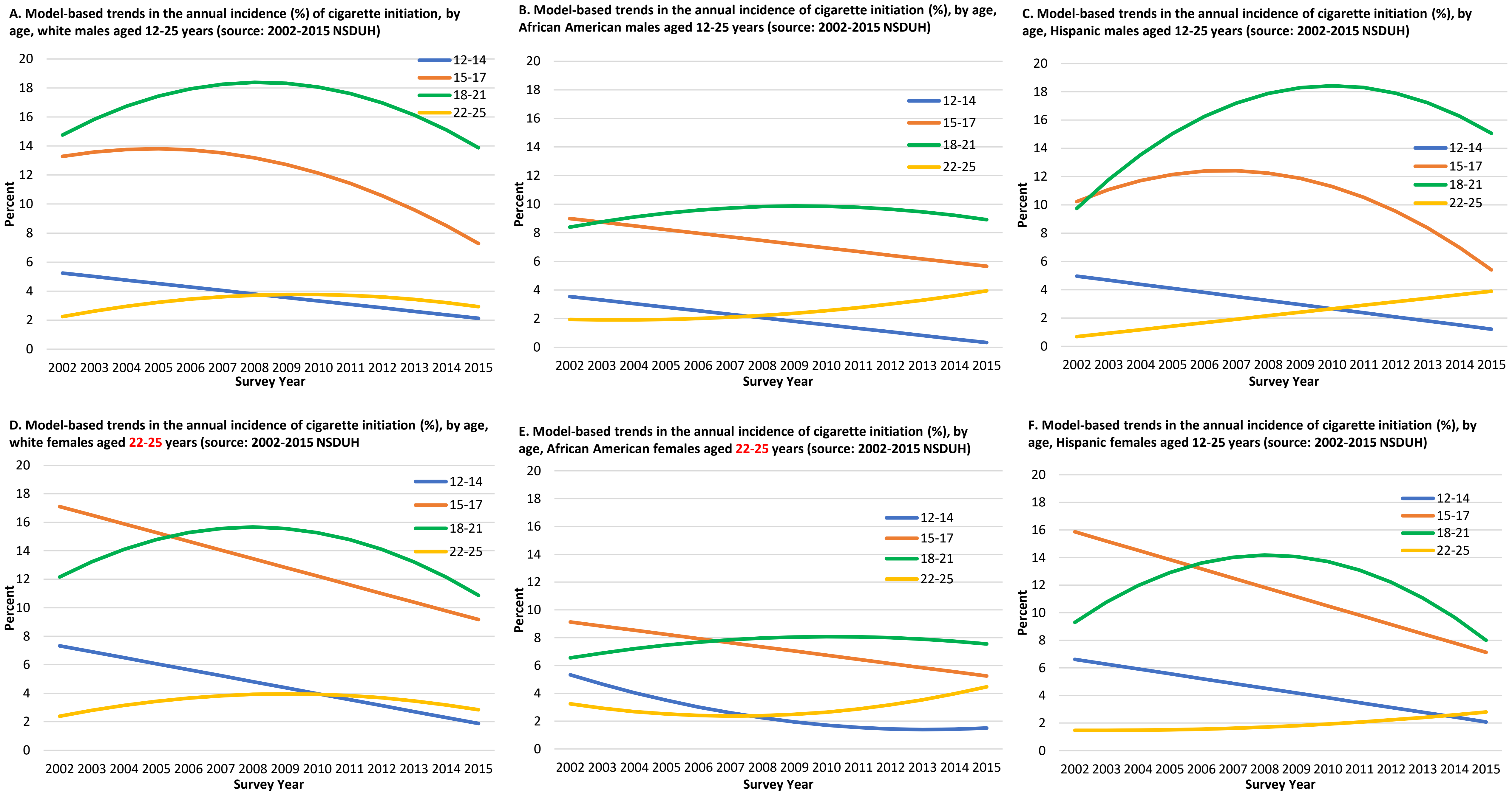

Supplement: S1 Fig — (PDF) [file pone.0200827.s001.pdf]
