# Supplementary material for: Patterns in first and daily cigarette initiation among youth and young adults from 2002 to 2015
Source: PLoS One. 2018 Aug 10;13(8):e0200827. doi: 10.1371/journal.pone.0200827 (PMC6086419; doi:10.1371/journal.pone.0200827)

S2 Fig. Model-based trends in the annual incidence of daily cigarette initiation (%), by age, gender, and race/ethnicity (source: 2002-2015 NSDUH)

A. Model-based trends in the annual incidence (%) of daily cigarette initiation, by age, white males aged 12-25 years (source: 2002-2015 NSDUH)

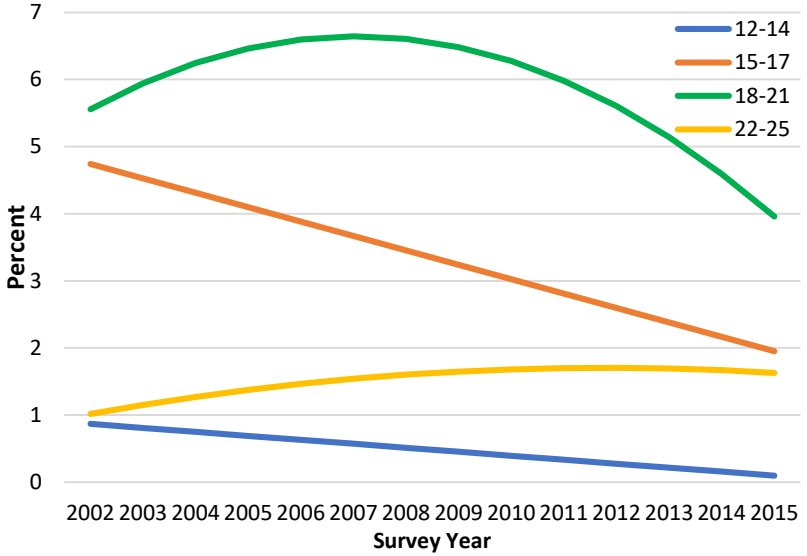

B. Model-based trends in the annual incidence (%) of daily cigarette initiation, by age, African American males aged 12-25 years (source: 2002-2015 NSDUH)

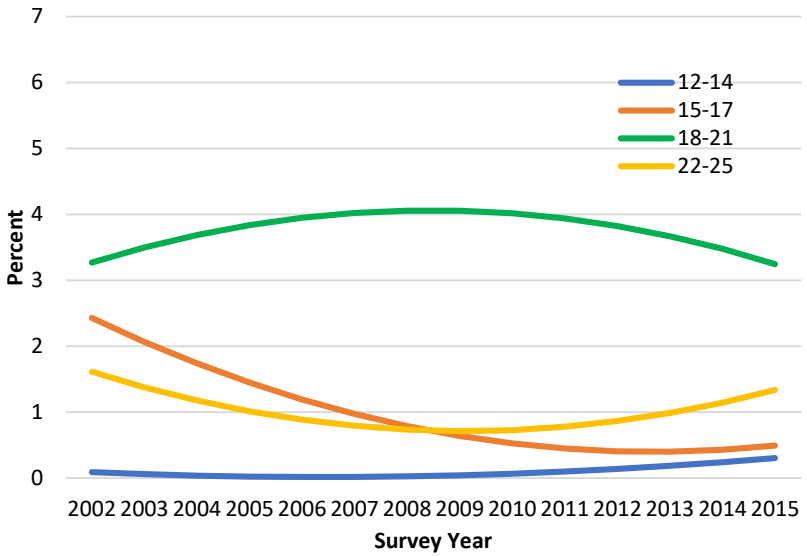

C. Model-based trends in the annual incidence (%) of daily cigarette initiation, by age, Hispanic males aged 12-25 years (source: 2002-2015 NSDUH)

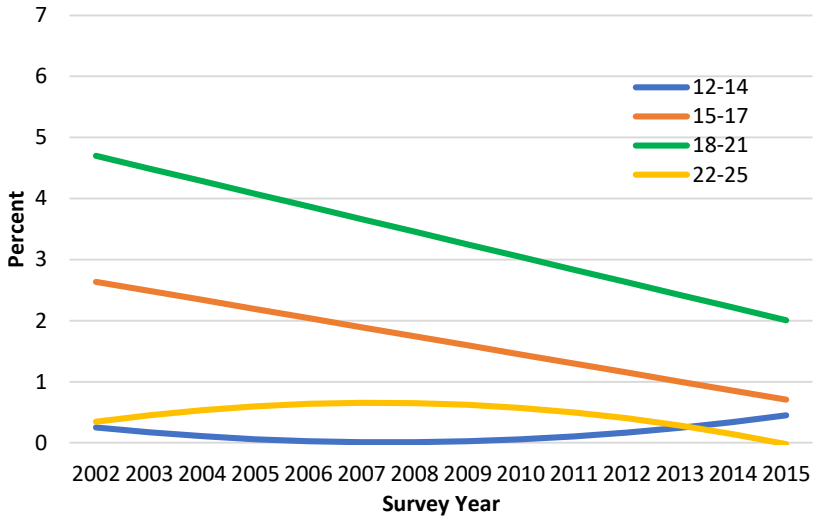

D. Model-based trends in the annual incidence (%) of daily cigarette initiation, by age, white females aged 12-25 years (source: 2002-2015 NSDUH)

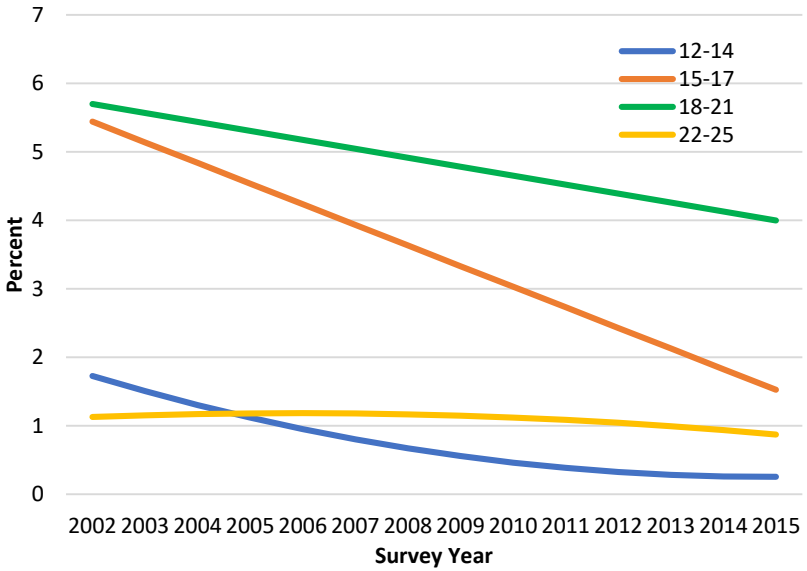

E. Model-based trends in the annual incidence (%) of daily cigarette initiation, by age, African American females aged 12-25 years (source: 2002-2015 NSDUH)

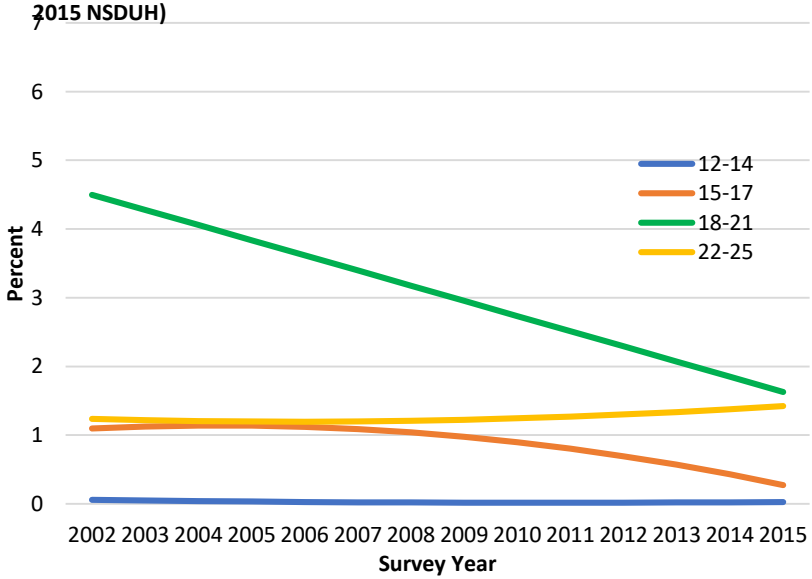

F. Model-based trends in the annual incidence (%) of daily cigarette initiation, by age, Hispanic females aged 12-25 years (source: 2002-2015 NSDUH)

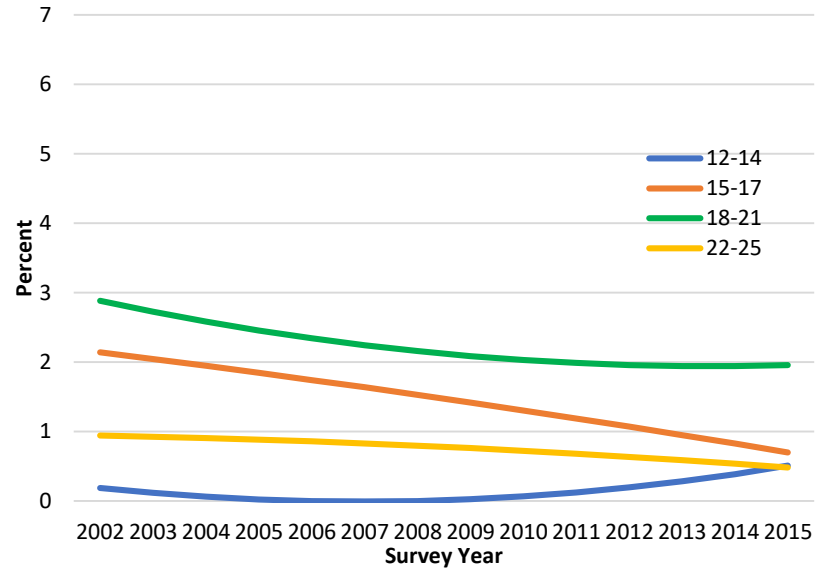

Supplement: S2 Fig — (PDF) [file pone.0200827.s002.pdf]
