# Supplementary material for: Patterns in first and daily cigarette initiation among youth and young adults from 2002 to 2015
Source: PLoS One. 2018 Aug 10;13(8):e0200827. doi: 10.1371/journal.pone.0200827 (PMC6086419; doi:10.1371/journal.pone.0200827)
Supplement: S1 Table — (PDF) [file pone.0200827.s009.pdf]

S1 Table. Autoregressive models of cigarette initiation and daily initiation among age, gender and racial/ethnic groups (source: 2002-2015 NSDUH).

			**Cigarette Initiation**		**Daily initiation**	
			**Odds ratio**	**P-value**	**Odds ratio**	**P-value**
Race/ethnicity	Gender	Age				
All Three Races	Overall	12-14	39.61	<0.01	1.03	0.56
		15-17	243.90	<0.01	5.08	<.01
		18-21	0.10	0.03	1.89	0.12
		22-25	0.41	<0.01	1.11	0.37
	Male	12-14	5.57	<0.01	1.05	0.70
		15-17	9.66	0.11	0.58	0.46
		18-21	0.01	<0.01	0.49	0.35
		22-25	0.19	<0.01	1.13	0.50
	Female	12-14	43.34	<0.01	0.96	0.54
		15-17	49.18	<0.01	5.07	<0.01
		18-21	0.24	0.30	1.22	0.67
		22-25	0.47	0.02	1.12	0.50
Non-Hispanic White	Overall	12-14	32.45	<0.01	3.05	<0.01
		15-17	572.98	<0.01	15.68	<0.01
		18-21	0.59	0.77	2.66	0.24
		22-25	1.94	0.28	1.09	0.76
Non-hispanic African American	Overall	12-14	10.77	0.11	0.88	0.23
		15-17	40.28	0.05	4.06	0.02
		18-21	0.38	0.72	2.06	0.54
		22-25	1.28	0.82	2.18	0.20
Hispanic	Overall	12-14	189.45	<0.01	1.55	0.20
		15-17	162.83	<0.01	13.60	<0.01
		18-21	0.61	0.86	2.66	0.43
		22-25	0.29	0.18	0.85	0.53
